# Suppression of double-stranded RNA sensing in cancer: molecular mechanisms and therapeutic potential

**DOI:** 10.1042/BST20230727

**Published:** 2024-09-02

**Authors:** Addison A. Young, Holly E. Bohlin, Jackson R. Pierce, Kyle A. Cottrell

**Affiliations:** Department of Biochemistry, Purdue University, West Lafayette, IN, U.S.A.

**Keywords:** autoimmunity, cancer, innate immunity, RNA editing, RNA-binding proteins, therapeutics

## Abstract

Immunotherapy has emerged as a therapeutic option for many cancers. For some tumors, immune checkpoint inhibitors show great efficacy in promoting anti-tumor immunity. However, not all tumors respond to immunotherapies. These tumors often exhibit reduced inflammation and are resistant to checkpoint inhibitors. Therapies that turn these ‘cold’ tumors ‘hot’ could improve the efficacy and applicability of checkpoint inhibitors, and in some cases may be sufficient on their own to promote anti-tumor immunity. One strategy to accomplish this goal is to activate innate immunity pathways within the tumor. Here we describe how this can be accomplished by activating double-stranded RNA (dsRNA) sensors. These sensors evolved to detect and respond to dsRNAs arising from viral infection but can also be activated by endogenous dsRNAs. A set of proteins, referred to as suppressors of dsRNA sensing, are responsible for preventing sensing ‘self’ dsRNA and activating innate immunity pathways. The mechanism of action of these suppressors falls into three categories: (1) Suppressors that affect mature RNAs through editing, degradation, restructuring, or binding. (2) Suppressors that affect RNA processing. (3) Suppressors that affect RNA expression. In this review we highlight suppressors that function through each mechanism, provide examples of the effects of disrupting those suppressors in cancer cell lines and tumors, and discuss the therapeutic potential of targeting these proteins and pathways.

## Introduction

In the 1890s Dr. William Coley unknowingly initiated the field of cancer immunotherapy when he treated a patient's tumor not with a pharmaceutical but with bacteria [1,2]. Coley later revised his treatment from live bacteria to a more refined agent derived from heat inactivated bacteria known as Coley's Toxins [1,3]. Coley's Toxins contained bacterial molecules that we now know of as pathogen associated molecular patterns (PAMPs). Most mammalian cells are capable of recognizing PAMPs via one of several pattern recognition receptors (PRRs) and can activate innate immunity pathways to fight off infection. It was likely this activity that was elicited by Coley's Toxins and thus promoted the immune system's ability to eradicate tumors. Today, Coley's Toxins have given way to many forms of cancer immunotherapy, including immune checkpoint inhibitors. Checkpoint inhibitors prevent the recognition of cancer cells as ‘self’ by cytotoxic T cells, thus enabling the killing of cancer cells [4]. These therapies have been used widely, and in many cases to significant effect. However, many tumors are resistant to checkpoint inhibitors [4]. Resistance can be overcome by activating PRRs and downstream innate immunity pathways to convert a ‘cold’ (immune-excluded) tumors into ‘hot’ (immune inflamed) tumors [5–11]. This review focuses on a specific set of PRRs — double-stranded RNA (dsRNA) sensors — and the therapeutic potential of activating those proteins to treat cancer.

### dsRNA sensors

**Figure 1.**
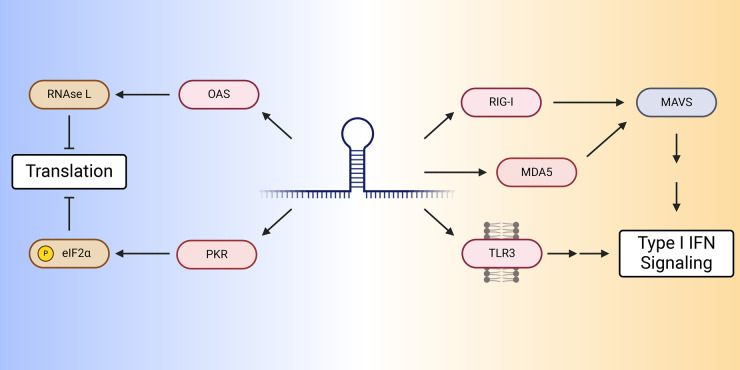
dsRNA sensing pathways. Double-stranded RNAs are detected by a number of dsRNA sensors that can trigger translational shutdown or induce IFN-I signaling. OAS detection of dsRNAs leads to the production of 2′,5′-oligoadenylate which activates RNase L, which degrades mRNA and rRNAs to shutdown translation. PKR activated by binding dsRNA phosphorylates eIF2α, which also shuts down translation. RIG-I and MDA5 detection of dsRNAs leads to the activation of MAVS, which activates downstream effectors that leads to IFN-I signaling. Similarly, TLR3 detection of dsRNAs also activates downstream effectors that lead to IFN-I. Created with BioRender.com.

Viral replication and transcription generates dsRNAs that can be sensed by an array of dsRNA sensors, Figure 1 [12,13]. The RIG-I like receptors (RLRs) are a class of dsRNA sensors and include RIG-I, MDA5 and LGP2 (which facilitates the activation of MDA5 and RIG-I) [14]. RIG-I and MDA5 function to sense dsRNA but have distinct mechanistic differences. RIG-I recognizes the 5′ terminus of dsRNAs and is specifically activated by RNAs with a 5′triphosphate — common to RNAs generated by viral polymerases [15]. RIG-I can be activated by relatively short dsRNAs, as short as 10–14 bp [16]. Conversely, MDA5 is typically only activated by longer dsRNAs, at least ∼500 bp, and has no specificity towards any 5′ structures [17]. The active forms of RIG-I and MDA5 have exposed caspase activation and recruitment domains that activate the adapter protein MAVS [14]. Activation of MAVS leads to a signaling cascade that drives a type I interferon (IFN-I) response including the expression of many interferon stimulated genes (ISGs).

Another key dsRNA sensor is protein kinase RNA-activated (PKR). Unlike MDA5 and RIG-I, PKR activates the integrated stress response pathway through phosphorylation of eIF2α which leads to global translational repression [18]. PKR is generally only activated by dsRNAs of at least 32 bp in length [19]. Interestingly, PKR is itself an ISG, and as such its expression is induced upon activation of MDA5 and RIG-I which likely enables infected cells to more robustly respond to viral infection.

In addition to the RLRs and PKR, two other groups of proteins have well characterized roles as dsRNA sensors: the oligoadenylate synthetases (OAS) and the toll-like receptor TLR3. Activation of TLR3 causes activation of the IFN-I pathway [20]. OAS proteins, when activated by dsRNA, generate 2′,5′-oligoadenylate which activates RNase L [21,22]. RNase L activation leads to global translational repression through degradation of mRNA and rRNA [22,23]. Together these dsRNA sensors provide an effective front line for the sensing of viral dsRNA and activation of an array of innate immunity pathways that help to fight infection, but as we describe below, these sensors also represent a vulnerability of cancer.

### dsRNA sensor agonists

Recently, it has become clear that activation of dsRNA sensors within tumors may be a viable therapeutic strategy for many cancers. A fitting example is the direct activation of RIG-I by short dsRNA agonists [6]. Because RIG-I can be activated by very short dsRNAs, it is highly amenable to activation by RNA agonists [16,24,25]. SLR14 is a highly potent RIG-I agonist that was shown to be effective in promoting anti-tumor immunity through activation of an IFN-I response in a mouse melanoma model [6]. Combination of SLR14 with the checkpoint inhibitor anti-PD-1 improved efficacy of the checkpoint inhibitor. These data highlight the potential of activating dsRNA sensors in cancer immunotherapy.

## Suppression of dsRNA sensing

**Figure 2.**
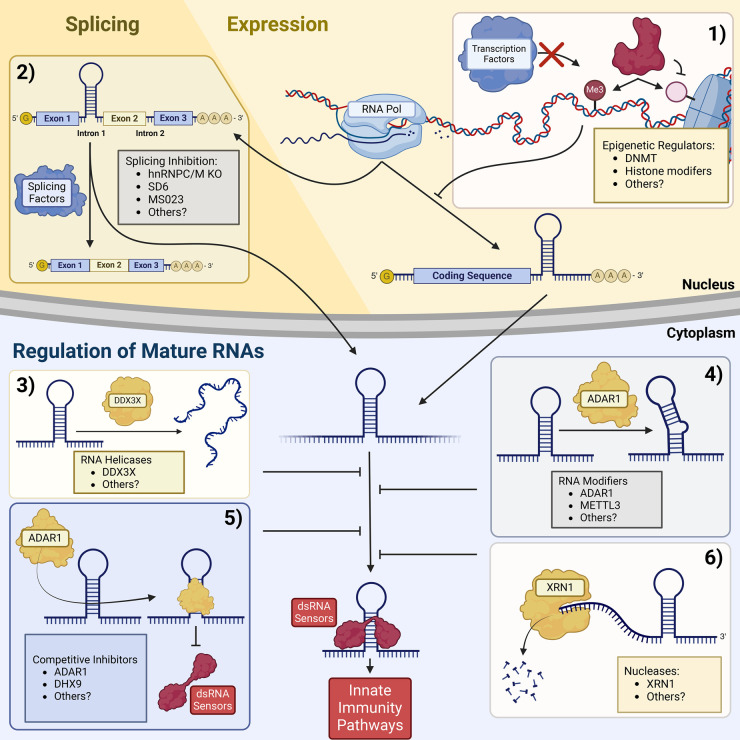
Mechanisms of suppression of dsRNA sensing. Double-stranded RNA detection is prevented through a number of mechanisms beginning at transcription. (1) Expression regulators such as DNMT, EZH2, or SUV39H1 can methylate DNA or modify histones in order to prevent the expression of mRNAs that contain retroelements that can form dsRNA. Pre-mRNAs can contain dsRNA regions within introns or 3′UTRs. (2) Splicing with the help of splicing factors like hnRNPC and hnRNPM can remove these introns containing dsRNA. Splicing failure in cancer or through spliceosome targeted therapies like SD6 or MS023 allow for dsRNA intronic regions to persist in mature mRNAs. RNAs with dsRNA regions in retained introns or 3′UTRs can still evade dsRNA sensors by multiple mechanisms of suppression of dsRNA sensing. (3) RNA helicases like DDX3X can unwind dsRNA regions. (4) RNA modifiers like ADAR1 can create structural changes that prevent dsRNA sensors from binding. (5) RNA binding proteins like cytoplasmic ADAR1 can prevent dsRNA sensors from binding dsRNAs through competitive inhibition. (6) Nucleases like XRN1 can degrade RNAs with double stranded regions. Failure to evade these dsRNA sensors results in activation of innate immunity pathways. Created with BioRender.com.

While direct activation of dsRNA sensors via RNAs may be efficacious, RNA agonists come with pharmacological challenges, for instance SLR14 had to be directly injected into the tumor [6]. Fortunately, exogenous RNAs aren't needed to activate dsRNA sensors. Viral dsRNA sensors in mammals have no sequence specificity [12]. This sequence-independent binding comes at a cost, since many of the sensors can be activated not only by viral dsRNA but also endogenous RNAs that form double-stranded regions (hereafter referred to as endogenous immunogenic dsRNAs). While the identity of endogenous immunogenic dsRNAs remains uncertain, there are many endogenous RNAs that form double-stranded regions, often through base pairing between inverted repeats of Alu elements or other endogenous retroelements — short interspersed nuclear elements, long interspersed nuclear elements, and others [12,26]. To prevent activation of dsRNA sensors by endogenous immunogenic dsRNAs, mammals have evolved several proteins and processes that suppress sensor activation through a variety of mechanisms. Below we highlight proteins that fall broadly into three mechanisms of suppression, Figure 2: suppressors that affect mature RNAs through modification, degradation, restructuring, or binding, suppressors that affect RNA processing, suppressors that affect RNA expression. Inhibition of these suppressors leads to activation of dsRNA sensors by endogenous immunogenic dsRNAs. This response is known as viral mimicry [27]. Like RIG-I agonists discussed above, inhibition or depletion of the suppressors of dsRNA sensing, and induction of viral mimicry, has the potential to promote anti-tumor immunity. As such, these suppressors of dsRNA sensing have become valuable cancer immunotherapy targets.

### Suppression through modification, degradation, restructuring, or binding of immunogenic RNAs

#### RNA modifiers

Adenosine deaminase acting on RNA 1 (ADAR1) is a prime example of a dsRNA binding protein that suppresses dsRNA sensing [28–30]. A-to-I editing by ADAR1 changes base-pairing (inosine prefers to pair with cytidine) and thus can impact mRNA coding, RNA structure, and/or regulation [31,32]. The only essential role for A-to-I editing by ADAR1 is suppression of MDA5 activation [28,29]. It is this function that when disrupted by mutations within ADAR1 causes Aicardi-Goutières syndrome, an autoimmune disorder characterized by persistent IFN-I signaling [33]. In mouse models, ADAR1 knockout is embryonic lethal, with increased IFN signaling and hematopoietic defects [34,35]. Double knockout of MAVS and ADAR1 in mouse models prevents embryonic lethality, but these mice die within a day of birth [36]. Similarly, catalytically inactive mutant ADAR1 mouse models (ADAR1^E861A/E861A^) are also embryonic lethal, with ADAR1^E861A/E861A^ MDA5^−/−^ mice surviving beyond weaning [29]. These data demonstrate that the deaminase activity of ADAR1 negatively regulates MDA5 activation and signaling through MAVS. Interestingly, knockout of the p150 isoform of ADAR1 closely phenocopies ADAR1 knockouts and can be rescued by knockout of MAVS, suggesting ADAR1p150 is the isoform primarily responsible for suppressing MDA5 activation [28,37]. Recent investigation into ADAR1 has established an explanation for the phenotypic differences between ADAR1^−/−^ MDA5^−/−^ and ADAR1^E861A/E861A^ MDA5^−/−^ mice. ADAR1^−/−^ MDA5^−/−^ mice show activation of PKR, which isn't observed in ADAR1^E861A/E861A^ MDA5^−/−^ mice [38]. PKR activation can be rescued in the absence of ADAR1 by expressing the dsRBDs of ADAR1, but not by expression of dsRNA binding deficient ADAR1. This trend can be replicated by using the dsRBDs of other dsRNA binding proteins. This discovery has led to the emergence of a dsRNA binding competition model, where ADAR1 suppresses PKR activation by competing with PKR for binding endogenous immunogenic dsRNAs [38]. Through these two mechanisms — A-to-I editing and binding of dsRNAs — ADAR1 is able to suppress dsRNA sensing and activation of innate immunity pathways.

The ability of ADAR1 to suppress dsRNA sensing can be exploited to kill cancer cells. Depletion of ADAR1 in many cancer cell lines, including those derived from breast, lung, liver and gastric cancers causes cell death and activation of dsRNA sensors [39–43]. Not all cell lines are sensitive to depletion of ADAR1. ADAR1-dependent cell lines, those that die upon depletion of ADAR1, tend to have a signature of elevated ISG expression driven by chronic activation of the cytosolic dsRNA sensing pathway of cGAS-STING [40,41,43]. Conversely, ADAR1-independent cell lines, those that are refractory to depletion of ADAR1, generally don't have this signature. Depletion of ADAR1 in ADAR1-dependent cells causes activation of MDA5, which drives IFN-I signaling, and PKR, with the latter largely contributing to cell death [40,41,43]. It has been proposed that ADAR1-dependent cells are more sensitive to depletion of ADAR1 due to elevated expression of MDA5 and PKR, both ISGs [41,43]. However, there are ADAR1-independent cell lines with low ISG expression, and further research is needed to understand why those cells are insensitive to depletion of ADAR1 [40].

While the cell intrinsic effects of depleting ADAR1 have therapeutic value, the cell extrinsic effects of targeting ADAR1 are likely to be more important in the clinic. Ishizuka et al. [5] used syngeneic mouse tumor models to show that ADAR1 knockout improved survivability and decreased tumor size. Combination of ADAR1 knockout with the immune checkpoint inhibitor anti-PD-1 improved survival and tumor clearance, even in an immunotherapy resistant B16 melanoma model. These effects were driven by activated IFN-I signaling and inflammation caused by knockout of ADAR1. These data highlight the potential of targeting ADAR1 to induce viral mimicry and enhance the efficacy of immunotherapies by turning ‘cold’ tumors ‘hot’.

ADAR1 isn't the only protein to suppress dsRNA sensing through RNA modification. The RNA methylating enzyme METTL3 is responsible for methylating adenosine within RNA to form N^6^-methyladenosine (m6A) which can influence the structure of RNAs, including reducing their propensity to form dsRNA [44–47]. Recently, it has been observed that inhibition of METTL3 in a mouse melanoma model causes accumulation of dsRNA and a viral mimicry phenotype [10]. Activation of multiple dsRNA sensors (PKR, MDA5, OAS-RNase L, and RIG-I) contributed to enhanced anti-tumor immunity upon inhibition of METTL3 in a mouse melanoma model. Like knockout of ADAR1, inhibition of METTL3 improves the efficacy of anti-PD1.

#### RNA helicases

Another key player in the suppression of dsRNA sensing in cancer is the DEAH-box helicase DHX9 [48,49]. DHX9 is a multifunctional protein, engaging in transcriptional and translational regulation, maintenance of genome integrity, microRNA biogenesis and many other cellular functions [50]. Like ADAR1, DHX9 contains dsRNA binding domains, which is unique amongst other DEAH box helicases in humans [48]. In cancer cells, DHX9 has been shown to suppress dsRNA sensing and activation of MDA5, PKR and RNase L, thereby blocking downstream innate immune responses [48,49]. As such, depletion of DHX9 in many cancer cells induces viral mimicry. In breast cancer cells, depletion of DHX9 resulted in reduced foci formation, increased apoptosis, and activation of PKR in most of the tested ADAR1-dependent cell lines [48]. In contrast, in ADAR1-independent cells, depletion of DHX9 alone had no effect on PKR activation. In those cells, combined depletion of DHX9 and ADAR1 was necessary to induce PKR activation. These data highlight a redundant role for DHX9 and ADAR1 in suppression of dsRNA sensing. Depletion of both ADAR1 and DHX9 also led to activation of IFN-I signaling and RNase L in one ADAR1-independent cell line. In SCLC cells, depletion of DHX9 alone caused MDA5 activation and activation of IFN-I signaling [49]. In that study it was also observed that depletion of DHX9 caused accumulation of R-Loops and DNA damage which contributed to IFN-I signaling through activation of cGAS-STING.

The mechanism of suppression of dsRNA sensing by DHX9 is still unclear. Murayama et al. [49] observed increased cytoplasmic dsRNA upon depletion of DHX9 and contributed this to loss of DHX9 helicase activity. Cottrell et al. [48] performed a rescue experiment in which expression of a helicase dead mutant of DHX9 (DHX9^K417R^) was shown to prevent activation of PKR in the absence of endogenous DHX9. Furthermore, expression of a truncated DHX9, with only its dsRBDs remaining, was sufficient to suppress activation of PKR, IFN-I and RNase L. These findings strongly support a helicase independent role for DHX9 in suppression of dsRNA sensing and is more consistent with DHX9 competing with dsRNA sensors for binding to endogenous immunogenic dsRNAs. Given the nuclear localization of DHX9, it may actually function through sequestering some of those dsRNAs in the nucleus, though further research is needed to evaluate this model.

While suppression of dsRNA sensing by DHX9 appears to be independent of its helicase activity, DDX3X is a DEAD-box helicase that uses its helicase domain to suppress dsRNA sensing [51,52]. Depletion of DDX3X results in accumulation of dsRNAs and activation of MDA5 and the IFN-I pathway in breast cancer cells [52]. These effects were phenocopied by an inhibitor of DDX3X and rescue experiments revealed that a helicase deficient DDX3X could not prevent increased ISG expression in DDX3X depleted cells. Like DHX9, co-depletion of ADAR1 and DDX3X further increased activation of dsRNA sensing pathways. The therapeutic potential of targeting DDX3X was highlighted in mouse studies in which depletion of DDX3X reduced tumor growth and promoted anti-tumor immunity.

#### RNA nucleases

Another approach to suppress dsRNA sensing is to degrade the dsRNAs. The highly conserved 5′-3′ exoribonuclease XRN1 was previously identified as codependent with ADAR1 (generally cell lines that are ADAR1-dependent are also XRN1-dependent) [53,54]. XRN1 binds dsRNA and plays a vital role in the regulation of RNA degradation [55,56]. Degradation of mRNA by XRN1 is important for mRNA turnover and gene regulation, and through this mechanism XRN1 influences how cells respond to other stresses [55,57]. XRN1's importance is highlighted by the fact that some viral infections reduce its activity in an effort to preserve viral dsRNA [58,59]. Recent studies targeting XRN1 in cancer cell lines have shown that depletion of XRN1 can induce a viral mimicry phenotype in cancer cells [53,54]. In XRN1-dependent cell lines, knockout of XRN1 caused reduced cell viability. Additionally, these cells exhibit increased cytosolic dsRNA and interferon signaling, and activation of PKR. Like ADAR1-dependency, some cell lines are refractory to depletion of XRN1 and don't produce a viral mimicry phenotype with loss of XRN1. Dependency on XRN1 appears to be influenced by baseline ISG expression and the expression of specific Alu elements [54]. XRN1-dependency can be induced by increasing dsRNA abundance or ISG expression. Treatment of XRN1-independent cells with palbociclib (a CDK4/6 inhibitor that induces IFN signaling through DNA damage [60]) or decitabine (a DNA methyltransferase (DNMT) inhibitor (DNMTi), discussed below) causes sensitivity to XRN1 depletion [54]. Thus, for some tumors, therapeutics that target XRN1 may need to be combined with other therapeutics, such as those above or ones that target another suppressor of dsRNA sensing.

### Suppression at the level of RNA processing

While the genome contains millions of copies of repetitive elements, many of those that are transcribed never make it to the cytoplasm because of splicing. Introns, which are generally much longer than exons, are common sites for editing by ADAR1 and thus contain double-stranded regions [31]. As we discuss below, when splicing is disrupted and introns are retained, the dsRNA within them can cause activation of dsRNA sensors.

#### hnRNPs

Heterogeneous nuclear ribonucleoproteins C and M (hnRNPC, hnRNPM) are RNA-binding proteins belonging to the hnRNP family and have important roles in cancer related to their functions in processing and splicing of RNAs [61–64]. Depletion of hnRNPC leads to reduced tumorigenesis via increased IFN-I signaling [65]. Likewise, tumors with low hnRNPM expression exhibit increased IFN-I signaling and, therefore, higher survival rates [66]. Depletion of both hnRNPC and hnRNPM causes splicing alterations referred to as cryptic splicing, which increases the abundance of cytoplasmic dsRNAs, including those arising from Alu elements [65,66]. In studies of both hnRNPC and hnRNPM, these cytoplasmic dsRNAs caused activation of dsRNA sensing pathways downstream of RIG-I or MDA5, resulting in IFN-I signaling [65–67]. Interestingly, depletion of hnRNPC and ADAR1 together, but neither alone, was shown to induce activation of the IFN-I pathway through MDA5 in THP-1 cells [67]. Herzner et al. suggest that this synergistic effect is caused by the combined effect of reduced A-to-I editing and accumulation of dsRNAs arising from retention of introns containing Alu elements.

#### Splicing inhibitors

Just as depletion of hnRNPC or hnRNPM leads to cell death due to sensing of endogenous dsRNAs arising from cryptic splicing, splicing inhibitors may reduce cancer viability through the same mechanism. Bowling et al. [68] found that the use of spliceosome-targeted therapies, such as spliceosome modulator sudemycin D6 (SD6), reduced triple-negative breast cancer (TNBC) viability by increasing relative dsRNA levels and causing activation of dsRNA sensors. Additionally, the group found a positive correlation between intron retention and inflammation in human primary breast tumors. Wu et al. [69] found that the use of MS023, a protein arginine methyltransferase (PRMT) inhibitor, induced a viral mimicry response due to intron retention and accumulation of cytosolic dsRNA. Like with ADAR1, elevated IFN signaling in TNBC cell lines correlated with susceptibility to MS023 treatment. Depletion of PRMT1 has also been shown to improve the efficacy of anti-PD-1 treatment in a melanoma mouse models [7].

### Suppression at the level of transcription

Suppression of dsRNA sensing can also be achieved at the level of transcription by epigenetic regulation via histone or DNA modifications. The role of epigenetic regulation in tumorigenesis and cancer progression is well documented and inhibition of epigenetic modifiers has great therapeutic potential [70]. DNA methylation of CpG dinucleotides by DNMTs has a well-established role in suppressing the expression of transposons, including retroelements that can produce endogenous immunogenic dsRNAs [27,70]. DNMTis, including azacitidine (5-azacytidine, 5-aza) and decitabine (5-aza-2′-deoxycytidine), induce de-methylation of promoter DNA, thereby increasing transcription of retroelements that form dsRNAs [27,71]. These FDA-approved DNMTi have been shown to induce a viral mimicry phenotype in several cancer types including non-small cell lung cancer as well as ovarian, breast and colorectal cancers [27,72–78]. The dsRNAs arising from derepressed retroelements are sensed by MDA5 and other dsRNA sensors leading to IFN-I signaling and other innate immune responses consistent with viral mimicry [77,79]. In some cases, DNMTi alone are insufficient to induce viral mimicry, but can induce viral mimicry when combined with depletion of ADAR1 [72,80]. As with depletion of other suppressors of dsRNA sensing, inhibition of DNMT by azacytidine improves the efficacy of the immune checkpoint inhibitor CTLA-4 [8].

Beyond CpG methylation, epigenetic repression of transposable element transcription also includes methylation of lysines on histone tails, such as Histone 3 lysine 27(H3K27) or lysine 9 (H3K9). Thus, activation of dsRNA sensing pathway can also be achieved by inhibition or deletion of multiple histone modifying enzymes (reviewed in [27]). For example, in prostate cancer and small cell lung cancer, inhibition of the H3K27 methyltransferase EZH2 promotes the expression of stimulated 3-prime antisense retroviral coding sequences (SPARCS), a subset of endogenous retroviruses (ERVs) silenced by H3K27 methylation [81]. The expression of SPARCS and other ERVs induce a viral mimicry phenotype through increased dsRNA expression and sensing leading to IFN-I pathway activation. Similarly, combined inhibition of DNMT and the H3K9 methyltransferase G9a with the inhibitor CM-272 increased dsRNAs, IFN-I signaling and improved efficacy of immune checkpoint inhibitors [82]. These findings highlight the importance of tightly controlled gene expression to limit dsRNA sensing.

### Knowledge gaps and challenges

While the data described above strongly support targeting suppressors of dsRNA sensing to treat various cancers, there are questions and challenges that remain that need to be addressed. The biggest challenge for harnessing many of the suppressors described here is a lack of inhibitors. For instance, there are currently no selective small molecule inhibitors of ADAR1, DHX9 or XRN1 available, though they may become widely available soon [83–86]. Identification of inhibitors needs to be informed by the mechanism of action of these suppressors. For ADAR1, an inhibitor of its deaminase activity may be beneficial to induce MDA5 activation, but since ADAR1 suppresses PKR activation through binding dsRNA, a deaminase inhibitor won't directly cause activation of PKR, which drives most cell death when ADAR1 is depleted [38,41]. Likewise, an inhibitor of DHX9 helicase activity is unlikely to cause activation of dsRNA sensors based on our current understanding of its mechanism of action [48]. It will also be important to carefully evaluate the toxicity of inhibitors that target suppressors of dsRNA sensing. While ADAR1, XRN1 and DDX3X are essential only in some cancer cell lines, DHX9 is more commonly essential across all cancer cell lines [87]. As such, drugs that target DHX9 may be more toxic than those targeting ADAR1, XRN1 or DDX3X. In all cases, the possibility remains that in some cell types or tissues, targeting suppressors of dsRNA sensing may lead to autoimmunity. How much this will affect the therapeutic potential of drugs that target suppressors of dsRNA sensing will remain unknown until selective inhibitors are identified and rigorously evaluated.

## Perspectives

- Preventing the detection of ‘self’ RNAs as foreign is essential for preventing autoimmunity. In cancer, disrupting the regulatory processes that prevent sensing of ‘self’ RNAs has great therapeutic potential.
- Suppressors of dsRNA sensing prevent activation of dsRNA sensors by endogenous immunogenic dsRNAs through multiple mechanisms. Therapies that target suppressors of dsRNA sensing have the potential to not only kill cancer cells directly, but to also promote anti-tumor immunity and the efficacy of cancer immunotherapies.
- The mechanisms of suppression employed by suppressors of dsRNA sensing need to be fully described at the molecular level to guide the development of therapies that target those proteins.

## Abbreviations

DNMT: DNA methyltransferase
DNMTi: DNA methyltransferase inhibitor
dsRNA: double-stranded RNA
ERV: endogenous retroviruse
IFN-I: type I interferon
ISG: interferon stimulated gene
OAS: oligoadenylate synthetases
PKR: protein kinase RNA
PRMT: protein arginine methyltransferase
PRR: pattern recognition receptor
RLR: RIG-I like receptor
SPARCS: stimulated 3-prime antisense retroviral coding sequences
TNBC: triple-negative breast cancer
